# Supplementary material for: Maternal HIV retesting during pregnancy and postpartum among high-risk populations in Kenya, South Africa, and Ukraine: Cost-effectiveness of preventing vertical transmission
Source: medRxiv. 2025 Apr 10:2025.04.08.25325413. Preprint. [Version 1] doi: 10.1101/2025.04.08.25325413 (PMC12036387; doi:10.1101/2025.04.08.25325413)
Supplement: Supplement 1 [file media-1.pdf]

Supplement: Cost-effectiveness of maternal HIV retesting during pregnancy and postpartum among high-risk populations in Kenya, South Africa, and Ukraine

Table of Contents

MODEL DESCRIPTION.......... 2

MODEL PARAMETERS 4

COST EFFECTIVENESS TABLES BY COUNTRY 5

SENSITIVITY ANALYSES.......... 8

Model description

We adapted a prior Markov model that was designed to measure the cost-effectiveness of HIV retesting during pregnancy and 12 months post-partum in Kenya, South Africa, and Ukraine. The full model details are published elsewhere.(1) Briefly, this is a deterministic state-transition model created using Excel software. There are 6 maternal HIV stages: 1 HIV negative, and 5 HIV positive states (acute HIV on ART, acute HIV not on ART, chronic HIV unknown ART status, chronic HIV on ART, chronic HIV not on ART). These 6 stages are stratified on 9 antenatal and postpartum periods – Stage 0: onset of pregnancy to first ANC; stage 1: first ANC to late ANC; stage 2: late ANC to delivery; stage 3: delivery; stage 4: first 6 weeks postpartum; stage 5: six to 14 weeks postpartum; stage 6: 14 weeks to six months postpartum; stage 7: six to nine months postpartum; stage 8: nine to 12 months postpartum. This results in a total of 54 states. Population size within each state represented in our model move between HIV negative to acute HIV positive and not on ART, and between HIV stages depending on our deterministic parameters.

Our model only includes pregnant members of key populations (FSW, PWID, HIV negative women in serodiscordant partnerships), and we estimate the number of individuals in each group using the estimated number of each key population per country and the fertility rate for each country. For our model parameters, we assume that key population parameters are the same as the general population parameters found in the original paper unless we found evidence that it differs. **Table S1** lists parameters that differ between our model and the original models. We assumed higher HIV prevalence among FSW (29%, 58%, and 5%) and among PWID (18%, 58%, and 31%) than the general population (6.1%, 31%, and 0.7%) in Kenya, South Africa, and Ukraine, respectively.(2–4) We also assumed higher HIV incidence among pregnant people in serodiscordant partnerships (7.02 per 100 PY in late pregnancy),(5) among FSW (4.7, 6.4, and 0.26 per 100 PY in Kenya, South Africa, and Ukraine respectively),(6,7) and among PWID (5.7, 6.4, and 0.35 per 100 PY in Kenya, South Africa, and Ukraine),(8,9) than the general population (1.18, 1.72, and 0.01 per 100 PY in Kenya, South Africa, and Ukraine).(5,10,11) Due to the absence of empirical HIV incidence rates for FSW in Ukraine we calculated

incidence based on the country's incidence:prevalence ratio and the prevalence of HIV among FSW. We assumed HIV prevalence and incidence among PWID were the same as FSW in South Africa.

Infant disability adjusted life-years (DALYs) and treatment costs were estimated over a 20-year time horizon and discounted at 3% annually. We model the number of infant infections but estimate cost and health events based on model outputs (infant infections). The use of pre-exposure prophylaxis (PrEP) is included in a sensitivity analysis, and we incorporate it into our model through reduced incidence of HIV.

Model parameters

Table S1. Key model parameters. We present only parameters that are different from the prior model. For the full list of prior model parameters see Meisner & Roberts et al. (2021). [1] Pregnant key population sizes were calculated using estimates of key population sizes and number of pregnancies per woman per country. [2] Maternal HIV incidence assumes zero risk of HIV during the period from delivery to 6 weeks postpartum. ‡ = same as non-key population for country; * = assumption. ANC=antenatal care, FSW=female sex workers, PWID=people who inject drugs, SD=Pregnant individuals in serodiscordant relationships, ART=antiretroviral therapy.

**Parameter**	**Kenya**			**South Africa**			**Ukraine**	
**Key population**	**FSW**	**PWID**	**SD**	**FSW**	**PWID**	**SD**	**FSW**	**PWID**
Population of pregnant individuals[1]	19,348 (12)	201 (2)	39,180 (13)	9,906 (14)	14,309 (15)	55,035 (16)	2,413 (17)	2,882 (18)
Percent of individuals who know HIV status at onset of pregnancy	0.68 (19)	0.94 (2)	0.96‡	0.81 (3)	0.80 (3)	0.94‡ (3)	0.58 (4)	0.43 (4)
HIV risk								
HIV prevalence	29.3% (2)	18.3% (2)	0*	57.7% (3)	57.7%*	0*	5.2% (20)	31.4% (4)
Maternal HIV incidence rate from pregnancy to 12 months postpartum (per 100 person-years)[2]	4.7 (6)	5.70 (8)	3.75 (5)	6.4 (6)	6.4*	3.75 (5)	0.26 (7)	0.35 (9)
Health care visits								
Attend first ANC	96%‡ (17)	96%‡ (17)	96%‡ (17)	90% (21)	94%‡ (22)	94%‡ (22)	99%‡ (23)	99%‡ (23)
First ANC (gestational age in weeks)	22‡ (17)	22‡ (17)	22‡ (17)	19 (21)	18‡ (16)	18‡ (16)	10*‡	10*‡
Antiretroviral coverage								
Maternal ART initiation	87%‡ (24)	87%‡ (24)	87%‡ (24)	94% (25)	87%‡ (24)	87%‡ (24)	95%‡ (7)	88% (26)
Viral suppression among those on ART	88%‡ (27)	88%‡ (27)	88%‡ (27)	72%‡ (28)	72%‡ (28)	72%‡ (28)	88%‡ (27)	88%‡ (27)
Weekly risk of ART dropout	0.56% (25)	0.56% (25)	0.56% (25)	0.56% (25)	0.56% (25)	0.56% (25)	0.56% (25)	0.56% (25)
Maternal mortality rate (per 100 person-years)								
During pregnancy	0.52‡ (29)	0.52‡ (29)	0.52‡ (29)	0.52‡ (29)	0.52‡ (29)	0.52‡ (29)	0.10‡ (29)	0.31 (30)
Delivery through 6 weeks postpartum	4.68‡ (31)	4.68‡ (31)	4.68‡ (31)	1.04‡ (31)	1.04‡ (31)	1.04‡ (31)	0.21‡ (31)	0.31 (30)
6 weeks to 12 months postpartum	0.52‡ (29)	0.52‡ (29)	0.52‡ (29)	0.52‡ (29)	0.52‡ (29)	0.52‡ (29)	0.10‡ (29)	1.30 (32)

Cost Effectiveness Tables by Country

Table S1. Cost-effectiveness of maternal HIV retesting scenarios for key populations in Ukraine. Late ANC is between 36-39 weeks of gestation. Testing offered at second ANC, or at delivery if not performed at late ANC, or at 6-week MCH visit if not performed at delivery or second ANC. [a]ICER=incremental cost effectiveness ratio, calculated as incremental costs (in 2023 US$) divided by DALYs averted compared with the next least-costly scenario. ANC=antenatal care; PP=postpartum; DALY=disability-adjusted life-year. All general population estimates are from Meisner & Roberts et al. (2021). Values in parentheses are negative, indicating that the scenario is cost-saving.

Retesting scenario	Infant infections	Total infections averted	% infections averted	Infant deaths	Total cost	Incremental cost	Total DALYs	Incremental DALYs averted	ICER
**Female sex workers (FSW)**									
No retesting	5	-	-	21	$462,767	-	408	-	-
Late ANC	5	1	12%	21	$466,054	$3,287	405	3	$1,010
Late ANC + 14 wk	5	1	13%	21	$477,982	$11,928	404	0	$27,626
Late ANC + 9 mo	5	1	13%	21	$478,420	$438	404	0	Dom
Late ANC + 6 mo	5	1	12%	21	$479,616	$1,196	404	0	Dom
Late ANC + 14 wk + 9 mo	5	1	14%	21	$489,536	$9,920	404	0	$57,949
Late ANC + 14 wk + 6 mo	5	1	14%	21	$489,809	$273	404	0	Dom
Late ANC + every 3 mo	5	1	14%	21	$500,893	$11,084	404	0	$126,960
**People who inject drugs (PWID)**									
No retesting	34	-	-	29	$2,937,316	-	629	-	-
Late ANC	32	3	8%	29	$2,908,757	-$28,559	615	14	($2,049)
Late ANC + 9 mo	31	3	8%	29	$2,919,370	$10,612	615	0	Dom
Late ANC + 14 wk	31	3	8%	29	$2,919,410	$40	615	0	$20,520
Late ANC + 6 mo	32	3	8%	29	$2,920,838	$1,428	615	0	Dom
Late ANC + 14 wk + 9 mo	31	3	8%	29	$2,929,074	$8,236	615	0	$82,419
Late ANC + 14 wk + 6 mo	31	3	8%	29	$2,929,376	$302	615	0	Dom
Late ANC + every 3 mo	31	3	8%	29	$2,938,744	$9,368	615	0	$198,210

Table S2. Cost-effectiveness of maternal HIV retesting scenarios for key populations in Kenya. Late ANC is between 36-39 weeks of gestation. Testing offered at second ANC, or at delivery if not performed at late ANC, or at 6-week MCH visit if not performed at delivery or second ANC. [a]ICER=incremental cost effectiveness ratio, calculated as incremental costs (in 2017 US$) divided by DALYs averted compared with the next least-costly scenario. ANC=antenatal care; PP=postpartum; DALY=disability-adjusted life-year. All general population estimates are from Meisner & Roberts et al. (2021). Values in parentheses are negative, indicating that the scenario is cost-saving.

Retesting scenario	Infant infections	Total infections averted	% infections averted	Infant deaths	Total cost	Incremental cost	Total DALYs	Incremental DALYs averted	ICER
**Female sex workers (FSW)**									
No retesting	638	-	-	810	$3,140,138	-	19,370	-	-
Late ANC	556	82	13%	797	$3,084,537	-$55,601	18,908	463	($120)
Late ANC +9mo	553	85	13%	796	$3,133,675	$49,138	18,889	19	Dom
Late ANC +14wk	549	89	14%	796	$3,134,479	$804	18,868	21	$1,268
Late ANC +6mo	552	86	13%	796	$3,142,423	$7,944	18,887	-18	Dom
Late ANC +14wk/9mo	547	91	14%	795	$3,176,636	$34,213	18,855	31	$3,144
Late ANC +14wk/6mo	547	91	14%	795	$3,179,443	$2,807	18,855	0	Dom
Late ANC +every 3mo	545	93	15%	795	$3,216,649	$37,206	18,846	9	$4,473
**People who inject drugs (PWID)**									
No retesting	4	-	-	8	$23,992	-	188	-	-
Late ANC	4	0	12%	8	$24,067	$76	185	3	$27
Late ANC +14wk	4	1	14%	8	$24,695	$628	185	0	$1,443
Late ANC +9mo	4	1	13%	8	$24,712	$17	185	0	Dom
Late ANC +6mo	4	1	13%	8	$24,815	$103	185	0	Dom
Late ANC +14wk/9mo	4	1	14%	8	$25,248	$433	184	0	$2,740
Late ANC +14wk/6mo	4	1	14%	8	$25,275	$28	184	0	Dom
Late ANC +every 3mo	4	1	15%	8	$25,748	$472	184	0	$3,686
**Serodiscordant couples**									
No retesting	468	-	-	1,504	$1,596,413	-	34,575	-	-
Late ANC	376	93	20%	1,489	$1,645,512	$49,099	34,052	523	$94
Late ANC +9mo	369	100	21%	1,488	$1,788,522	$143,010	34,011	41	Dom
Late ANC +14wk	358	111	24%	1,486	$1,795,569	$7,047	33,950	61	$1,473
Late ANC +6mo	365	104	22%	1,487	$1,819,843	$24,274	33,990	-40	Dom
Late ANC +14wk/9mo	353	115	25%	1,485	$1,916,918	$97,074	33,923	68	Dom
Late ANC +14wk/6mo	351	118	25%	1,485	$1,930,447	$13,530	33,910	13	$3,379
Late ANC +every 3mo	348	120	26%	1,485	$2,034,520	$104,073	33,895	15	$6,715

Table S3. Cost-effectiveness of maternal HIV retesting scenarios for key populations in South Africa. Late ANC is between 36-39 weeks of gestation. Testing offered at second ANC, or at delivery if not performed at late ANC, or at 6-week MCH visit if not performed at delivery or second ANC. [a]ICER=incremental cost effectiveness ratio, calculated as incremental costs (in 2017 US$) divided by DALYs averted compared with the next least-costly scenario. ANC=antenatal care; PP=postpartum; DALY=disability-adjusted life-year. All general population estimates are from Meisner & Roberts et al. (2021). Values in parentheses are negative, indicating that the scenario is cost-saving.

Retesting scenario	Infant infections	Total infections averted	% infections averted	Infant deaths	Total cost	Incremental cost	Total DALYs	Incremental DALYs averted	ICER
**Female sex workers (FSW)**									
No retesting	401		-	395	$2,755,966	-	9,036	-	-
Late ANC	365	36	6%	389	$2,782,571	$26,605	8,836	199	$134
Late ANC + 9mo	364	37	6%	389	$2,814,819	$32,248	8,832	4	Dom
Late ANC + 14wk	363	37	6%	389	$2,821,108	$6,290	8,825	8	$3,333
Late ANC + 6mo	364	38	6%	389	$2,831,010	$9,902	8,830	-5	Dom
Late ANC + 14 wk + 9mo	362	39	7%	389	$2,849,540	$18,530	8,822	8	$9,399
Late ANC + 14 wk + 6mo	362	38	7%	389	$2,860,970	$11,430	8,821	1	$10,940
Late ANC + every 3 mo	362	39	7%	389	$2,885,935	$24,966	8,819	2	$12,448
**People who inject drugs (PWID)**									
No retesting	587	-	-	572	$3,853,344	-	13,094	-	-
Late ANC	545	46	8%	565	$3,891,390	$38,046	12,861	233	$163
Late ANC + 9mo	544	47	8%	565	$3,934,998	$43,609	12,856	5	Dom
Late ANC + 14wk	542	47	8%	565	$3,943,232	$8,234	12,847	9	$3,852
Late ANC + 6mo	543	49	8%	565	$3,955,899	$12,667	12,854	-6	Dom
Late ANC + 14 wk + 9mo	542	50	8%	564	$3,982,636	$26,737	12,844	10	$11,430
Late ANC + 14 wk + 6mo	541	50	8%	564	$3,998,350	$15,713	12,843	1	$13,407
Late ANC + every 3 mo	541	50	9%	564	$4,033,953	$35,603	12,840	2	$15,642
**Serodiscordant couples**									
No retesting	641	-	-	1940	$2,212,997	-	41,380	-	-
Late ANC	486	154	24%	1,915	$2,676,441	$463,444	40,521	859	$540
Late ANC + 9mo	482	159	25%	1,914	$3,047,403	$370,962	40,495	27	Dom
Late ANC + 14wk	469	171	27%	1,913	$3,119,904	$72,501	40,428	67	$4,734
Late ANC + 6mo	476	164	26%	1,914	$3,231,467	$111,563	40,467	-39	Dom
Late ANC + 14 wk + 9mo	466	175	27%	1,912	$3,462,528	$231,061	40,409	57	Dom
Late ANC + 14 wk + 6mo	463	178	28%	1,911	$3,611,918	$149,390	40,391	19	$13,195
Late ANC + every 3 mo	461	180	28%	1,911	$3,924,983	$313,065	40,381	9	$33,293

Sensitivity analyses

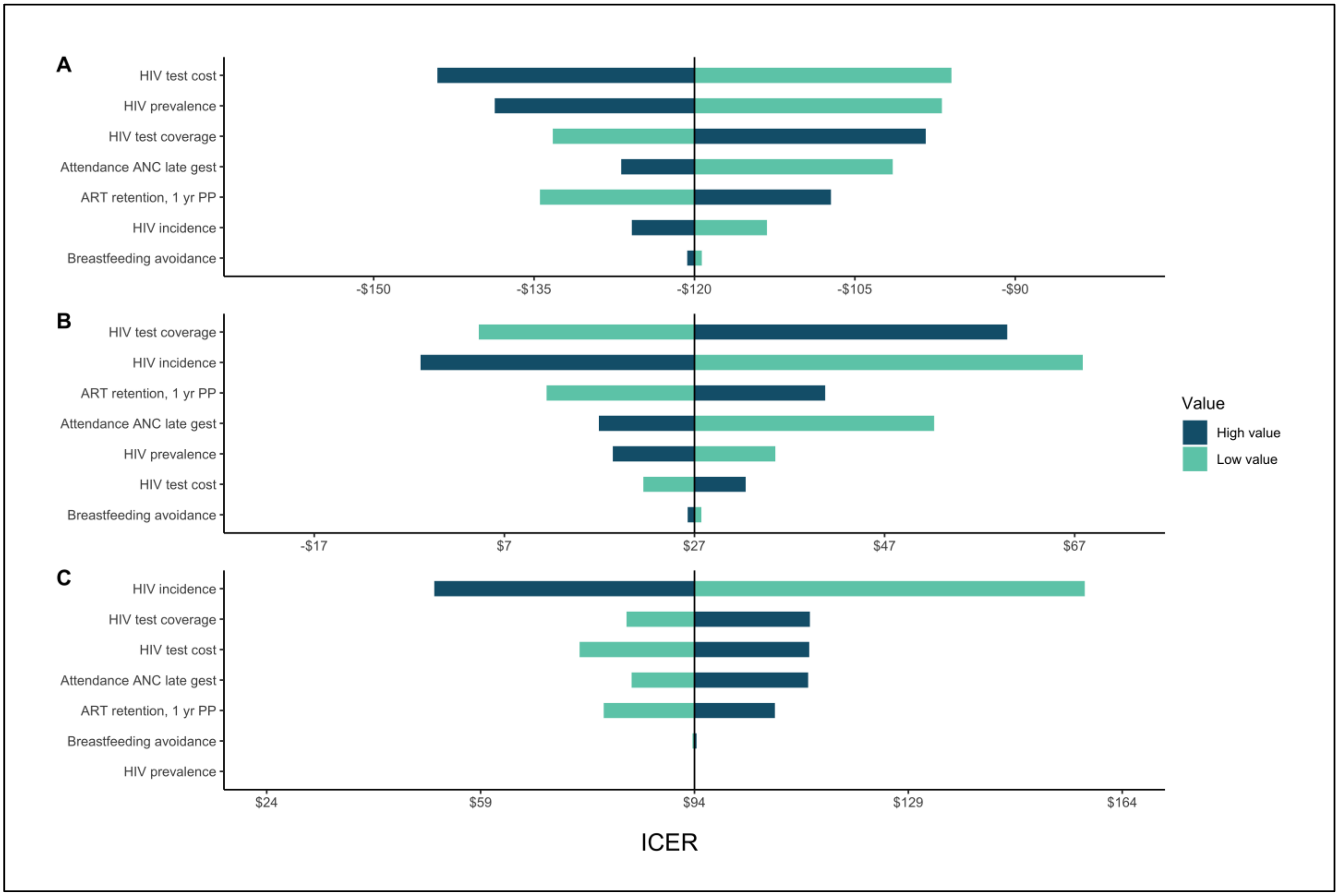

Figure S1. One-way sensitivity analysis for Kenya late ANC retesting: (A) FSW, (B) PWID, (C) Serodiscordant couples. Each model parameter (y-axis) was individually adjusted 20% higher (High value) and 20% lower (low value). The x-axis shows the change in ICER from the unadjusted value. Each population is centered on the unadjusted ICER. Note that x-axes are at different scales. ANC = Antenatal care

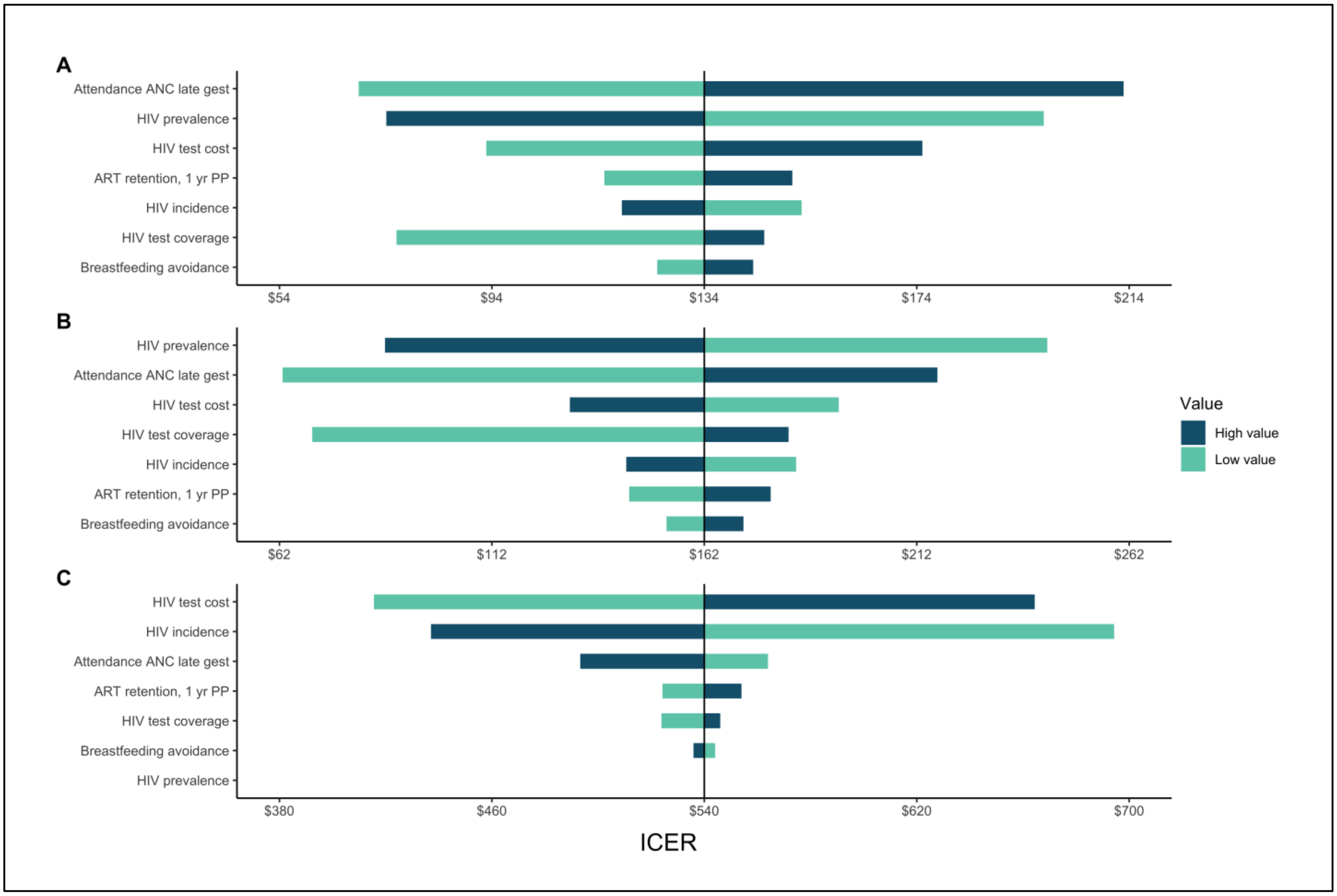

Figure S2. One-way sensitivity analysis for South Africa late ANC retesting: (A) FSW, (B) PWID, (C) Serodiscordant couples. Each model parameter (y-axis) was individually adjusted 20% higher (High value) and 20% lower (low value). The x-axis shows the change in ICER from the unadjusted value. Each population is centered on the unadjusted ICER. Note that x-axes are at different scales. ANC = Antenatal care

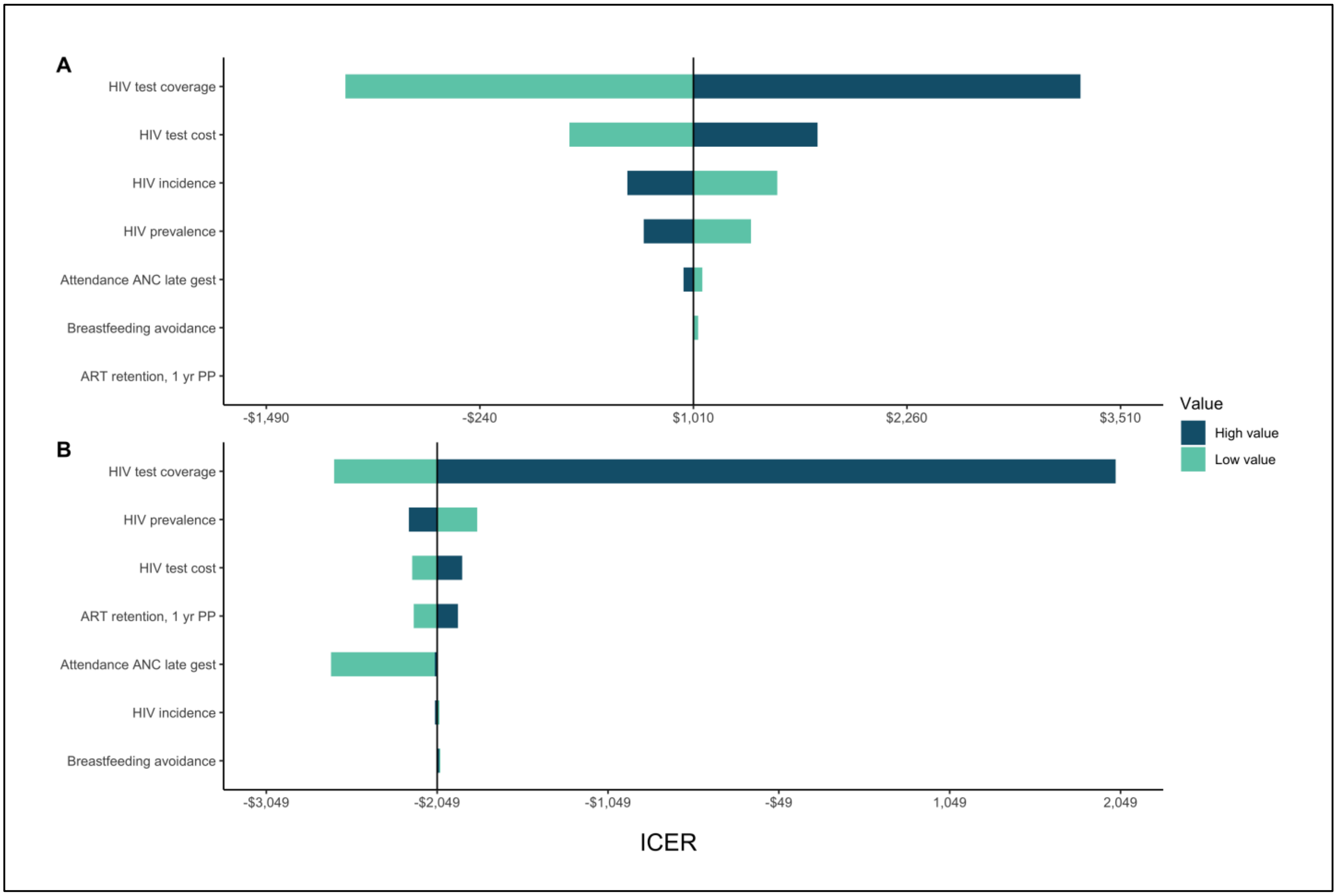

Figure S3. One-way sensitivity analysis for Ukraine late ANC retesting: (A) FSW, (B) PWID. Each model parameter (y-axis) was individually adjusted 20% higher (High value) and 20% lower (low value). The x-axis shows the change in ICER from the unadjusted value. Each population is centered on the unadjusted ICER. Note that x-axes are at different scales. ANC = Antenatal care

Table S4. Cost-effectiveness of maternal HIV retesting scenarios for key populations in Ukraine: sensitivity analysis including 20% decreases in HIV incidence and prevalence, and 15% PrEP uptake. Late ANC is between 36-39 weeks of gestation. Testing offered at second ANC, or at delivery if not performed at late ANC, or at 6-week MCH visit if not performed at delivery or second ANC. [a]ICER=incremental cost effectiveness ratio, calculated as incremental costs (in 2023 US$) divided by DALYs averted compared with the next least-costly scenario. ANC=antenatal care; PP=postpartum; DALY=disability-adjusted life-year. All general population estimates are from Meisner & Roberts et al. (2021). Values in parentheses are negative, indicating that the scenario is cost-saving.

Retesting scenario	Infant infections	Total infections averted	% infections averted	Infant deaths	Total cost	Incremental cost	Total DALYs	Incremental DALYs averted	ICER
**Female sex workers (FSW)**									
No retesting	5	-	-	21	$1,210,247	-	407	-	-
Late ANC	5	1	12%	21	$1,214,013	$3,766	404	3	$1,229
Late ANC + 14 wk	5	1	13%	21	$1,225,811	$11,798	404	0	$30,313
Late ANC + 9 mo	5	1	13%	21	$1,226,220	$409	404	0	Dom
Late ANC + 6 mo	5	1	13%	21	$1,227,310	$1,090	404	0	Dom
Late ANC + 14 wk + 9 mo	5	1	14%	21	$1,237,285	$9,976	404	0	$64,310
Late ANC + 14 wk + 6 mo	5	1	14%	21	$1,237,543	$258	404	0	Dom
Late ANC + every 3 mo	5	1	14%	21	$1,248,598	$11,055	403	0	$141,361
**People who inject drugs (PWID)**									
No retesting	34	-	-	29	$3,578,237	-	629	-	-
Late ANC	31	3	8%	29	$3,550,023	-$28,214	615	14	($2,044)
Late ANC + 9 mo	31	3	8%	29	$3,560,543	$10,520	615	0	Dom
Late ANC + 14 wk	31	3	8%	29	$3,560,595	$52	614	0	$21,588
Late ANC + 6 mo	31	3	8%	29	$3,561,939	$1,344	615	0	Dom
Late ANC + 14 wk + 9 mo	31	3	8%	29	$3,570,217	$8,279	614	0	$91,214
Late ANC + 14 wk + 6 mo	31	3	8%	29	$3,570,508	$290	614	0	Dom
Late ANC + every 3 mo	31	3	8%	29	$3,579,860	$9,353	614	0	$220,855

Table S5. Cost-effectiveness of maternal HIV retesting scenarios for key populations in Kenya: sensitivity analysis including 20% decreases in HIV incidence and prevalence, and 15% PrEP uptake. Late ANC is between 36-39 weeks of gestation. Testing offered at second ANC, or at delivery if not performed at late ANC, or at 6-week MCH visit if not performed at delivery or second ANC. [a]ICER=incremental cost effectiveness ratio, calculated as incremental costs (in 2017 US$) divided by DALYs averted compared with the next least-costly scenario. ANC=antenatal care; PP=postpartum; DALY=disability-adjusted life-year. All general population estimates are from Meisner & Roberts et al. (2021). Values in parentheses are negative, indicating that the scenario is cost-saving.

Retesting scenario	Infant infections	Total infections averted	% infections averted	Infant deaths	Total cost	Incremental cost	Total DALYs	Incremental DALYs averted	ICER
**Female sex workers (FSW)**									
No retesting	593	-	-	803	$4,385,208	-	19,115	-	-
Late ANC	518	74	13%	790	$4,338,338	-$46,871	18,695	420	($112)
Late ANC +9mo	516	77	13%	790	$4,383,789	$45,452	18,681	14	Dom
Late ANC +14wk	513	80	13%	789	$4,385,283	$1,493	18,663	17	$1,476
Late ANC +6mo	516	77	13%	790	$4,391,055	$5,773	18,678	-15	Dom
Late ANC +14wk/9mo	511	82	14%	789	$4,425,058	$34,003	18,653	25	$3,991
Late ANC +14wk/6mo	511	82	14%	789	$4,427,486	$2,428	18,653	0	Dom
Late ANC +every 3mo	510	83	14%	789	$4,463,489	$36,003	18,646	7	$5,755
**People who inject drugs (PWID)**									
No retesting	4	-	-	8	$39,789	-	187	-	-
Late ANC	4	0	11%	8	$39,894	$106	184	3	$41
Late ANC +14wk	4	1	13%	8	$40,497	$603	184	0	$1,533
Late ANC +9mo	4	1	12%	8	$40,510	$13	184	0	Dom
Late ANC +6mo	4	1	12%	8	$40,604	$94	184	0	Dom
Late ANC +14wk/9mo	3	1	14%	8	$41,031	$427	184	0	$2,934
Late ANC +14wk/6mo	3	1	14%	8	$41,056	$26	184	0	Dom
Late ANC +every 3mo	3	1	14%	8	$41,517	$461	183	0	$3,983
**Serodiscordant couples**									
No retesting	425	-	-	1,497	$5,429,103	-	34,332	-	-
Late ANC	341	84	20%	1,483	$5,484,704	$55,601	33,858	473	$117
Late ANC +9mo	335	90	21%	1,482	$5,622,472	$137,768	33,822	37	Dom
Late ANC +14wk	325	100	24%	1,481	$5,628,709	$6,237	33,767	55	$1,572
Late ANC +6mo	332	94	22%	1,482	$5,650,266	$21,557	33,803	-36	Dom
Late ANC +14wk/9mo	321	104	25%	1,480	$5,747,007	$96,740	33,742	61	Dom
Late ANC +14wk/6mo	319	106	25%	1,480	$5,758,859	$11,852	33,731	11	$3,627
Late ANC +every 3mo	316	109	26%	1,479	$5,861,683	$102,824	33,717	14	$7,376

Table S6. Cost-effectiveness of maternal HIV retesting scenarios for key populations in South Africa: sensitivity analysis including 20% decreases in HIV incidence and prevalence, and 15% PrEP uptake. Late ANC is between 36-39 weeks of gestation. Testing offered at second ANC, or at delivery if not performed at late ANC, or at 6-week MCH visit if not performed at delivery or second ANC. [a]ICER=incremental cost effectiveness ratio, calculated as incremental costs (in 2017 US$) divided by DALYs averted compared with the next least-costly scenario. ANC=antenatal care; PP=postpartum; DALY=disability-adjusted life-year. All general population estimates are from Meisner & Roberts et al. (2021). Values in parentheses are negative, indicating that the scenario is cost-saving.

Retesting scenario	Infant infections	Total infections averted	% infections averted	Infant deaths	Total cost	Incremental cost	Total DALYs	Incremental DALYs averted	ICER
**Female sex workers (FSW)**									
No retesting	383	-	-	392	$3,120,617	-	8,937	-	-
Late ANC	351	36	6%	387	$3,148,859	$28,242	8,758	179	$158
Late ANC + 9mo	350	38	7%	387	$3,179,280	$30,421	8,755	3	Dom
Late ANC + 14wk	349	37	6%	387	$3,185,629	$6,349	8,749	6	$3,903
Late ANC + 6mo	350	37	6%	387	$3,194,241	$8,612	8,753	-5	Dom
Late ANC + 14 wk + 9mo	349	38	7%	387	$3,212,909	$18,668	8,747	7	$12,036
Late ANC + 14 wk + 6mo	349	39	7%	387	$3,223,949	$11,040	8,746	1	$13,623
Late ANC + every 3 mo	348	39	7%	387	$3,248,560	$24,612	8,744	1	$16,586
**People who inject drugs (PWID)**									
No retesting	581	-	-	571	$4,439,821	-	13,061	-	-
Late ANC	540	46	8%	564	$4,478,396	$38,575	12,835	226	$171
Late ANC + 9mo	539	47	8%	564	$4,521,151	$42,754	12,831	4	Dom
Late ANC + 14wk	538	47	8%	564	$4,529,425	$8,274	12,822	8	$4,062
Late ANC + 6mo	539	49	8%	564	$4,541,519	$12,094	12,828	-6	Dom
Late ANC + 14 wk + 9mo	537	50	9%	564	$4,568,242	$26,723	12,819	9	$12,425
Late ANC + 14 wk + 6mo	537	50	9%	564	$4,583,756	$15,514	12,818	1	$14,419
Late ANC + every 3 mo	537	50	9%	564	$4,619,101	$35,345	12,816	2	$17,214
**Serodiscordant couples**									
No retesting	574		-	1929	$7,607,327		41,007		-
Late ANC	434	139	24%	1,907	$8,076,000	$468,673	40,233	774	$606
Late ANC + 9mo	431	143	25%	1,906	$8,434,616	$358,616	40,212	22	Dom
Late ANC + 14wk	420	153	27%	1,905	$8,507,021	$72,405	40,154	58	$5,432
Late ANC + 6mo	427	147	26%	1,906	$8,606,414	$99,393	40,190	-36	Dom
Late ANC + 14 wk + 9mo	418	156	27%	1,904	$8,841,382	$234,969	40,139	51	Dom
Late ANC + 14 wk + 6mo	415	159	28%	1,904	$8,983,480	$142,098	40,126	14	$16,834
Late ANC + every 3 mo	414	160	28%	1,904	$9,294,850	$311,370	40,118	8	$41,068

References

1. Meisner J, Roberts DA, Rodriguez P, Sharma M, Newman Owiredu M, Gomez B, et al. Optimizing HIV retesting during pregnancy and postpartum in four countries: a cost-effectiveness analysis. J Int AIDS Soc. 2021 Apr 31;24(4).
2. UNAIDS Kenya Country Factsheet. 2018.
3. UNAIDS. UNAIDS South Africa Country Factsheet. Unaids. 2018;1–6.
4. UNAIDS. Ukraine Country Factsheet. 2019.
5. Thomson KA, Hughes J, Baeten JM, John-Stewart G, Celum C, Cohen CR, et al. Increased Risk of HIV Acquisition Among Women Throughout Pregnancy and During the Postpartum Period: A Prospective Per-Coital-Act Analysis Among Women With HIV-Infected Partners. J Infect Dis. 2018;218:16–25.
6. Birdthistle I, Tanton C, Tomita A, de Graaf K, Schaffnit SB, Tanser F, et al. Recent levels and trends in HIV incidence rates among adolescent girls and young women in ten high-prevalence African countries: a systematic review and meta-analysis. Lancet Glob Health. 2019;7(11).
7. Joint UNAIDS. UNAIDS Data 2019 [Internet]. Vol. 268, Science. 2019 [cited 2022 Dec 26]. Available from: https://www.unaids.org/sites/default/files/media_asset/2019-UNAIDS-data_en.pdf
8. Kurth AE, Cleland CM, Des Jarlais DC, Musyoki H, Lizcano JA, Chhun N, et al. HIV prevalence, estimated incidence, and risk behaviors among people who inject drugs in Kenya. J Acquir Immune Defic Syndr (1988). 2015;70(4):420–7.
9. Simmons R, Malyuta R, Chentsova N, Karnets I, Murphy G, Medoeva A, et al. HIV incidence estimates using the limiting antigen avidity EIA assay at testing sites in Kiev City, Ukraine: 2013-2014. PLoS One. 2016;11(6):2013–4.
10. Kinuthia J, Drake AL, Matemo D, Richardson BA, Zeh C, Osborn L, et al. HIV acquisition during pregnancy and postpartum is associated with genital infections and partnership characteristics. AIDS. 2015;29:2025–33.
11. Ishikawa N, Dalal S, Johnson C, Hogan DR, Shimbo T, Shaffer N, et al. Should HIV testing for all pregnant women continue? Cost-effectiveness of universal antenatal testing compared to focused approaches across high to very low HIV prevalence settings. J Int AIDS Soc. 2016;19(1):21212.
12. National AIDS Control Council (Kenya), National AIDS and STI Control Programme (NASCOP) (Kenya), United Nations Office on Drugs and Crime (UNODC), University of California San Francisco. Kenya Most At Risk Populations Size Estimate Consensus Report. 2013.
13. National AIDS and STI Control Programme (NASCOP) K. Kenya AIDS Indicator Survey 2012: Final Report. Nairobi, NASCOP June 2014 [Internet]. 2014;66:S3–12. Available from: http://content.wkhealth.com/linkback/openurl?sid=WKPTLP:landingpage&an=00126334-201405011-00002
14. Stacey M, Konstant T, Rangasami J, Stewart M, Mans G. Estimating the size of the sex worker population in South Africa, 2013. 2013. 1–40 p.
15. Peltzer K, Phaswana-Mafuya N. Drug use among youth and adults in a population-based survey in South Africa. South African Journal of Psychiatry. 2018;24(1):1–6.
16. DHS Program. South Africa Demographics and Health Survey 2016. 2016.
17. Kenya National Bureau of Statistics. Kenya Demographic and Health Survey 2014. Annals of human biology. 2015.
18. UNODC. Number and prevalence of PWID and those living with HIV among this group [Internet]. 2017. Available from: https://dataunodc.un.org/drugs/pwid_hiv-2017
19. Agot K, Cain M, Medley A, Kimani J, Gichangi P, Kiio C, et al. Formative assessment to identify perceived benefits and barriers of HIV oral self-testing among female sex workers, service providers, outreach workers, and peer educators to inform scale-up in Kenya. AIDS Care - Psychological and Socio-Medical Aspects of AIDS/HIV. 2022;34(6).
20. UNAIDS. UNAIDS Ukraine Country Factsheet [Internet]. 2017. Available from: https://www.unaids.org/en/regionscountries/countries/ukraine

21. Parmley L, Rao A, Kose Z, Lambert A, Max R, Phaswana-Mafuya N, et al. Antenatal care presentation and engagement in the context of sex work: Exploring barriers to care for sex worker mothers in South Africa. Reprod Health. 2019;16(Suppl 1):1–12.
22. Myer L, Phillips T, Manuelli V, McIntyre J, Bekker LG, Abrams EJ. Evolution of antiretroviral therapy services for HIV-infected pregnant women in Cape Town, South Africa. Journal of Acquired Immune Deficiency Syndrome. 2015;
23. World Health Organization RO for E. Report on 2018–2019 pre-validation assessment of elimination of mother-to-child transmission of HIV and syphilis in Ukraine [Internet]. 2020 [cited 2022 Dec 27]. Available from: https://apps.who.int/iris/handle/10665/336180
24. Bhattacharjee P, Musyoki HK, Becker M, Musimbi J, Kaosa S, Kioko J, et al. HIV prevention programme cascades: insights from HIV programme monitoring for female sex workers in Kenya. J Int AIDS Soc. 2019;22(S4).
25. Eakle R, Gomez GB, Naicker N, Bothma R, Mbogua J, Cabrera Escobar MA, et al. HIV pre-exposure prophylaxis and early antiretroviral treatment among female sex workers in South Africa: Results from a prospective observational demonstration project. PLoS Med. 2017;14(11):1–17.
26. Thorne C, Semenenko I, Malyuta R. Prevention of mother-to-child transmission of human immunodeficiency virus among pregnant women using injecting drugs in Ukraine, 2000-10. Addiction. 2012;
27. Snippenburg W, Nellen F, Smit C, Wensing A, Godfried MH, Mudrikova T. Factors associated with time to achieve an undetectable HIV RNA viral load after start of antiretroviral treatment in HIV-1-infected pregnant women. J Virus Erad. 2017;3:34–9.
28. Brittain K, Mellins CA, Remien RH, Phillips TK, Zerbe A, Abrams EJ, et al. Impact of HIV-status disclosure on HIV viral load in pregnant and postpartum women on antiretroviral therapy. JAIDS Journal of Acquired Immune Deficiency Syndromes. 2019;1.
29. World Health Organization (WHO). WHO Life Tables by Country [Internet]. 2020 [cited 2022 Dec 27]. Available from: https://apps.who.int/gho/data/node.main.LIFECOUNTRY?lang=en
30. Cirelli JF, Surita FG, Costa ML, Parpinelli MA, Haddad SM, Cecatti JG. The burden of indirect causes of maternal morbidity andmortality in the process of obstetric transition: A cross-sectional multicenter study [A importância das causas indiretas da morbidade e mortalidade maternas no processo de transição obstétrica: Um es. Revista Brasileira de Ginecologia e Obstetricia [Internet]. 2018;40(3):106–14. Available from: https://www.scopus.com/inward/record.uri?eid=2-s2.0-85048337906&doi=10.1055%2Fs-0038-1623511&partnerID=40&md5=db9c2b7c7eb8c7af4bf69a52603459b7
31. World Bank. Maternal mortality ratio [Internet]. 2019 [cited 2022 Dec 27]. Available from: https://data.worldbank.org/indicator/sh.sta.mmrt
32. Mathers BM, Degenhardt L, Bucello C, Lemon J, Wiessing L, Hickman M. Mortality among people who inject drugs: a systematic review and meta-analysis. Bull world Health Organ. 2013;(91):102–23.
